# The association between *CCR5* Δ32 polymorphism and susceptibility to breast cancer

**DOI:** 10.18632/oncotarget.19959

**Published:** 2017-08-05

**Authors:** Junlong Li, Yuan Peng, Hui Liu, Qiang Wu

**Affiliations:** ^1^ Department of Medicine and Teaching, Southwest Hospital, Third Military Medical University, Chongqing 400038, China; ^2^ Department of Oncology, Southwest Hospital, Third Military Medical University, Chongqing 400038, China; ^3^ Department of Hepatobiliary Surgery, Southwest Hospital, Third Military Medical University, Chongqing 400038, China

**Keywords:** CCR5, polymorphism, breast cancer, susceptibility, meta-analysis

## Abstract

**Background:**

Chemokine C-C motif receptor 5 (*CCR5*) gene polymorphisms have been proposed to play important roles in tumors. Δ32 polymorphism of this gene might correlate with breast cancer (BC) susceptibility. Nevertheless, inconsistent conclusions have been achieved as yet. We carried out this meta-analysis to draw a more comprehensive and convincing conclusion on this issue.

**Results:**

No significant correlation of *CCR5* Δ32 polymorphism with individual susceptibility to BC was detected in either total analysis (Δ32 vs. WT: OR=1.12, 95% CI=0.76-1.65; WT/Δ32 vs. WT/WT: OR=1.21, 95% CI=0.81-1.80) or subgroup analyses by ethnicity and control source.

**Methods:**

All eligible studies were searched from electronic databases including Chinese National Knowledge Infrastructure (CNKI), PubMed, EMBASE, and Google Scholar Web. Strength of association between *CCR5* Δ32 polymorphism and BC susceptibility was evaluated using pooled odds ratios (ORs) with their corresponding 95% confidence intervals (95% CIs). To further detect their correlation in specific populations, subgroup analyses were performed based on ethnicity and control source. Sensitivity analysis was conducted in this meta-analysis to test statistical stability of the final results. Publication bias among included studies was inspected with Begg’s funnel plot and Egger’s test.

**Conclusion:**

*CCR5* Δ32 polymorphism may not independently affect the risk of BC.

## INTRODUCTION

Breast cancer (BC) is the most frequently diagnosed malignancy among women around the world, and its incidence rate has been increasing globally, especially in developed countries compared with developing ones [1, 2]. With the advancements in medical techniques, the mortality rate of this cancer shows a downward trend in the past few decades [3], though its morbidity sees no decline [4]. As a leading cause of cancer-related deaths in females [5], BC has higher morbidity and mortality in the worldwide in recent years [6]. According to corresponding statistics, about 50% of the new BC cases and 60% of BC-related deaths occur in less developed countries and regions, and the incidence age of such cancer shows an increasing tendency in younger women [7]. Both genetic and epigenetic factors are reported to be involved in the etiology of this complex and multi-factorial disease [8]. Epidemiological studies have also shown that female reproductive status plays vital roles in the initiation of BC [9]. However, among women facing same risk factors for BC, only a small part finally develop this cancer, and familial history of such malignancy is reported to contribute to approximately 5% to 10% of all BC cases [10], which indicates the significant functions of genetic factors in BC onset [11].

Chemokines are a class of small cytokines which could be categorized into four subfamilies, namely the CXC, CC, C and CX3C chemokines. Chemokines take part in immune cell trafficking during inflammatory responses through interacting with chemokine receptors on the surface of immune cells [12, 13]. As a member of the CC chemokine receptor group, chemokine C-C motif receptor 5 (CCR5) can regulate the trafficking and effector functions of memory/effector T cells, macrophages, immature dendritic cells and natural killer cells [14]. This receptor binds to the chemokines MIP-1α (CCL3), MIP-1β (CCL4), and RANTES (CCL5), and exerts its functions through G protein [15]. In addition, CCR5 also plays a pivotal role in cancer progression via recruiting immune cells [16]. In *CCR5* gene, some polymorphisms have been identified, and among them, the Δ32 polymorphism has been reported to affect the severity of multiple autoimmune and infectious diseases through mediating inflammatory responses [17].

The influence of *CCR5* Δ32 polymorphism on BC susceptibility has been explored in previous studies among different populations, but the number of these researches was relatively small. We therefore performed this meta-analysis to more systematically explore this issue.

## RESULTS

### Study characteristics

**Figure 1:**
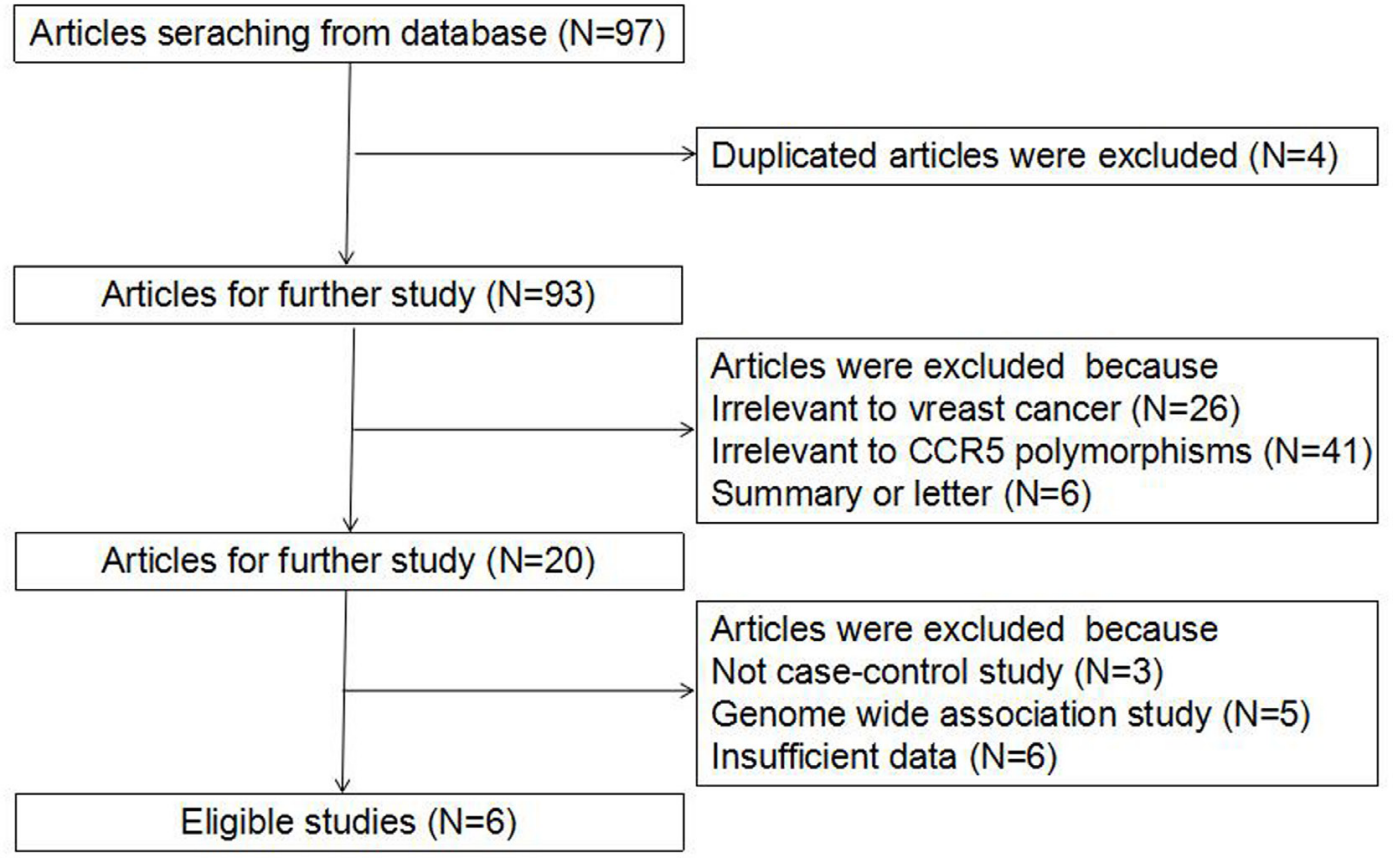
Flow diagram for the process of literature selection

Literature search strategy initially identified 97 potentially relevant publications from the databases, and 4 of them were firstly removed for being duplicates (Figure 1). During further screening, 87 more papers were excluded for irrelevance with BC (26) or *CCR5* Δ32 polymorphism (41), being summaries or letters (6) or genome wide association studies (5), without a case-control design (3) or having usable data (6). Ultimately, a total of 6 eligible studies recruiting 1839 participants were incorporated into the present meta-analysis [18–23]. Among them, 3 were carried out among Caucasians, 2 were among Asians and 1 was among Brazilians. Table 1 describes the major characteristics of all included studies.

**Table 1:** Principal information of included studies in the meta-analysis

First author	Year	Country	Ethnicity	Control source	Genotyping method	Genotype and allele distribution (Case/Control)			
						WT/WT	WT/Δ32	WT	Δ32
Aoki	2009	Brazil	Caucasian	population	PCR	67/77	5/12	139/166	5/14
Zafiropoulos	2004	Greece	Caucasian	population	PCR	238/196	26/14	502/406	26/14
Degerli	2005	Turkey	Caucasian	hospital	PCR	36/254	3/13	75/521	3/13
Eskandari-Nasab	2014	Iran	Asian	population	PCR	231/196	5/7	467/399	5/7
Guleria	2012	India	Asian	population	PCR	76/80	4/0	156/160	4/0
Banin-Hirata	2015	Brazil	Brazilian	population	PCR	107/167	11/12	225/346	11/14

Notes: WT/WT, wild-type homozygous genotype; WT/Δ32, heterozygous genotype; WT, wild-type allele; Δ32, mutated allele; PCR, polymerase chain reaction.

### Data synthesis

**Figure 2:**
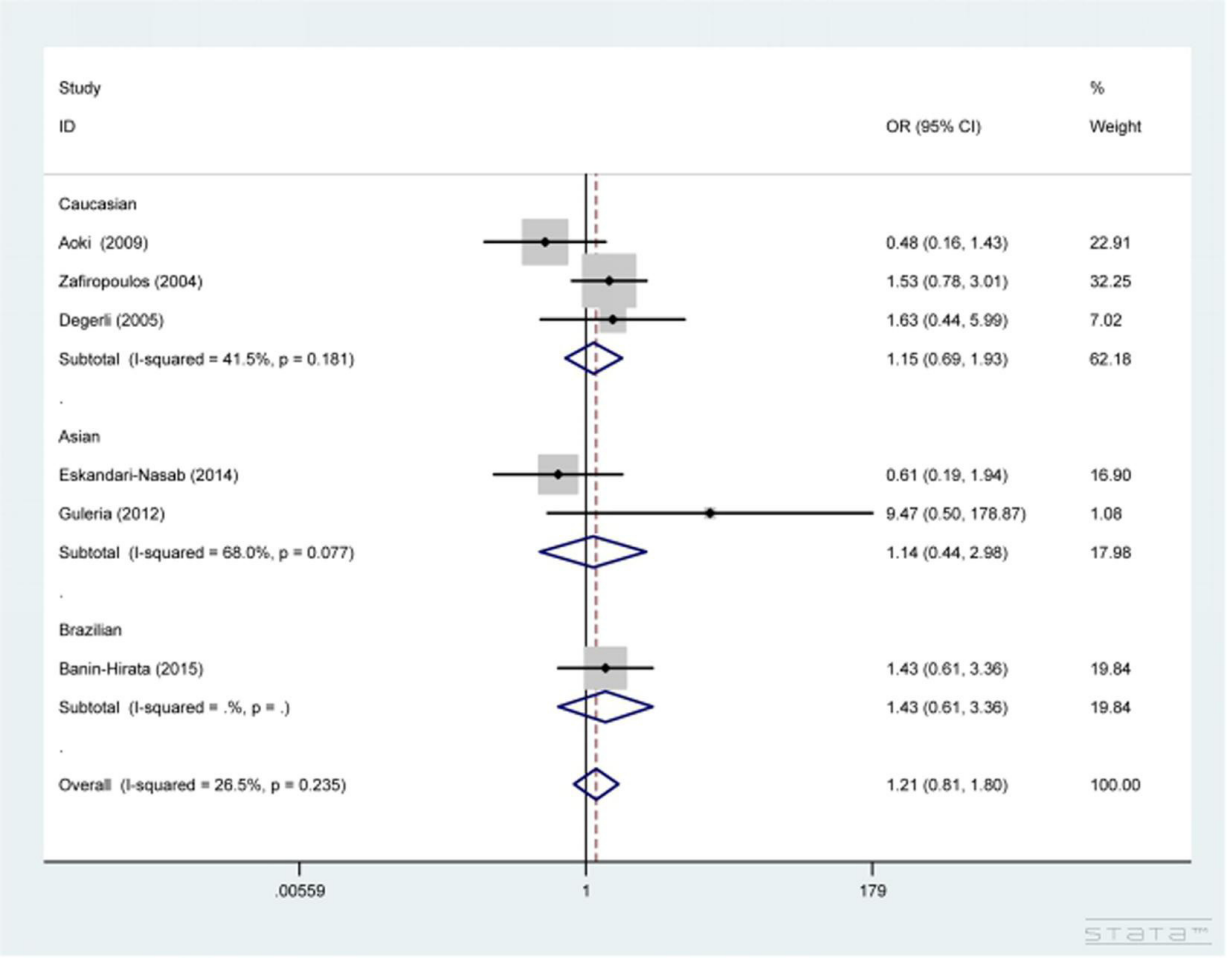
Forest plot for the association between CCR5 Δ32 polymorphism and the susceptibility to breast cancer under WT/Δ32 vs WT/WT contrast. The squares and horizontal lines correspond to the study-specific OR and 95% CI. The area of the squares reflects the weight (inverse of the variance). The diamond represents the summary OR and 95% CI.

As shown in Table 2, *CCR5* Δ32 polymorphism showed no correlation with the risk of BC under the genetic models of Δ32 vs. WT and WT/Δ32 vs. WT/WT (OR=1.12, 95% CI=0.76-1.65; OR=1.21, 95% CI=0.81-1.80 (Figure 2)). After stratification analyses by ethnicity and source of control, a similar trend was also revealed in Asian, Caucasian, Brazilian, hospital-based and population-based subgroups.

**Table 2:** *CCR5* Δ32 polymorphism and the susceptibility to breast cancer

Group	No. of studies	Odds ratio (95% confidence interval) / *P* value for heterogeneity			
		Δ32 vs. WT		WT/Δ32 vs. WT/WT	
Caucasian	3	1.08 (0.66, 1.78)	0.114	1.15 (0.69, 1.93)	0.181
Asian	2	1.14 (0.44, 2.96)	0.079	1.14 (0.44, 2.98)	0.077
Brazilian	1	1.21 (0.54, 2.71)	/	1.43 (0.61, 3.36)	/
Population	5	1.08 (0.72, 1.62)	0.133	1.17 (0.77, 1.78)	0.161
Hospital	1	1.60 (0.45, 5.76)	/	1.63 (0.44, 5.99)	/
Total	6	1.12 (0.76, 1.65)	0.192	1.21 (0.81, 1.80)	0.235

Notes: WT/WT, wild-type homozygous genotype; WT/Δ32, heterozygous genotype; WT, wild-type allele; Δ32, mutated allele.

### Heterogeneity test

Q test revealed no significant heterogeneity among included studies (*P*>0.05), so the fixed-effects model was employed for ORs calculation.

### Sensitivity analysis

The deletion of any one of the included studies did not alter summary ORs qualitatively during the whole process of sensitivity analysis (data not shown), confirming the statistical stability and robustness of our findings.

### Publication bias examination

**Figure 3:**
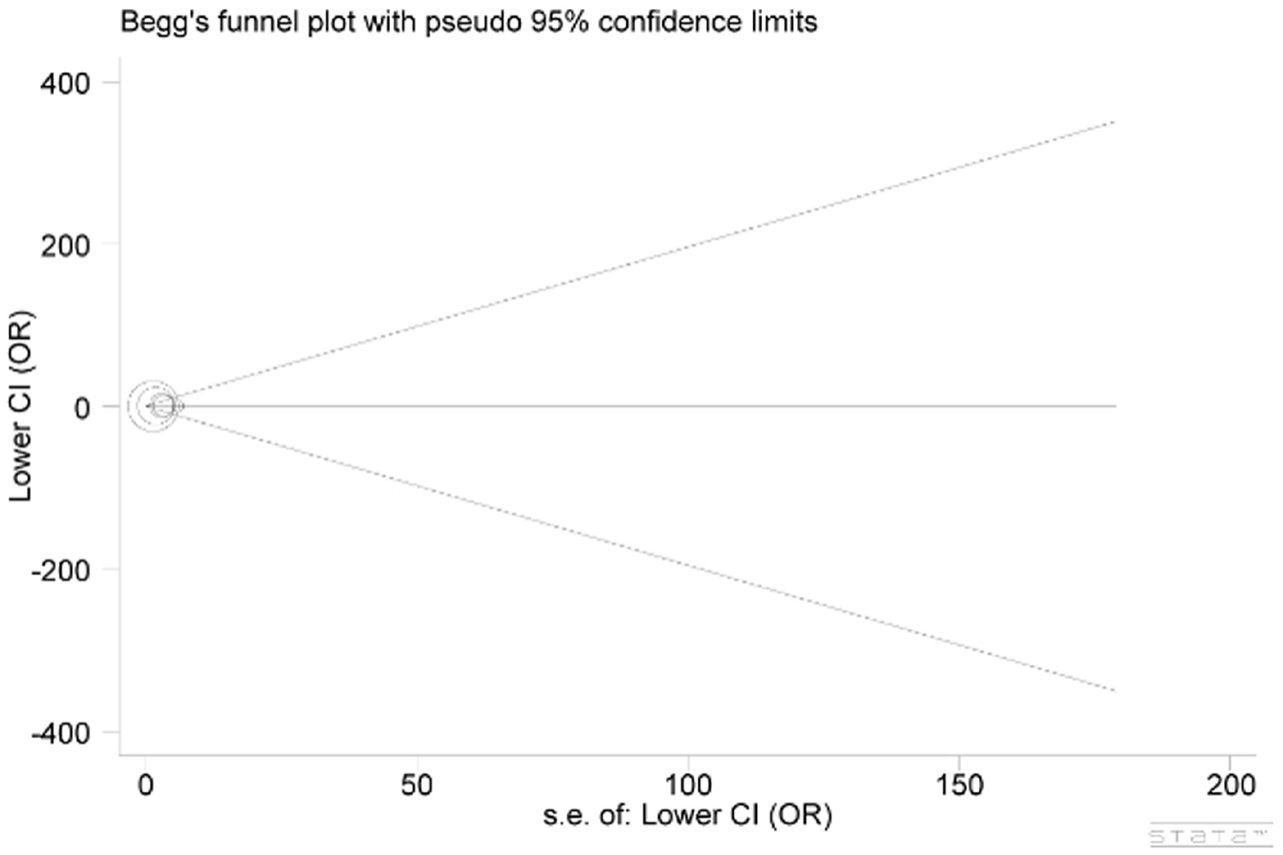
Begg’s funnel plot for publication bias Each point represents a separate study for the indicated association. Log(OR), natural logarithm of OR. Horizontal line, mean effect size.

In the investigation of potential publication bias, none of the funnel plots was found to be asymmetrical through visual check (Figure 3), and these conditions were statistically verified by evidence from Egger’s test (*P*=0.307), which demonstrated that the publication bias was negligible.

## DISCUSSION

In spite the pathogenesis of BC is not definite, documents demonstrate that various factors participate in the onset of BC [24–26]. Besides, increasing evidences manifest that the genetic backgrounds may decide the individual susceptibility to BC development.

CCR5 mainly distributed in various immune cells and endothelial cells, and is regarded as the specific surface marker for Th1 cells [27]. After combining with its specific ligands (such as CCL3, CCL4 and CCL5), CCR5 can activate G protein and ultimately elevate the intracellular concentration of Ca^2+^ and activate protein kinases C, thus regulating the migration and proliferation of immune cells [28]. The Δ32 polymorphism, one of the most studied polymorphisms in this gene, refers to the mutation caused by the missing of 32 bases in the gene. The Δ32 polymorphism in CCR5 gene may lead to abnormal protein expression. It has been investigated that this polymorphism might participate in the occurrence of various cancers, including BC. In a study performed among a Turkish population, the heterozygote genotype of Δ32 polymorphism was revealed to be an independent risk factor for breast cancer development [21]. A similar meta-analysis exploring the association between CCR5 Δ32 polymorphism and various cancer types also suggested a possible impact thereof on BC [29]. However, this association was not suggested by Aoki et al., Zafiropoulos et al., and Banin-Hirata et al. in their studies [18–20]. Considering the small sample sizes in some of them, we performed this meta-analysis for a more reliable conclusion on this relationship.

In this meta-analysis, we statistically synthesized findings from several eligible case-control studies involving the association between *CCR5* Δ32 polymorphism and BC risk, and found no evidence for significant correlation between them in both the overall analysis and subgroup analyses according to ethnicity and control source. As could be known, our conclusions were inconsistent from those of some previous studies. Reasons for the discrepancy may include the following aspects. To begin with, the selection criteria of participants were not the same, so other potential risk factors for BC such as ethnicity and BC family history might statistically differ. Next, the sample size of some studies was relatively smaller, which might weaken the statistical power of final results. Also, in some studies, the Δ32 polymorphism had interactive effects on BC together with other genetic variants, which meant that those variants might interfere with the independent role of Δ32 polymorphism. This conclusion was contrary to that from a previous meta-analysis by Lee et al. [29], in which a positive relationship of the polymorphism with the risk of BC was revealed. As for the discrepancies between our findings and their conclusion, they might be explained by some differences between our meta-analysis and their study. For example, our meta-analysis incorporated more studies on BC compared to theirs. Additionally, the participants for BC in their study were all Caucasians while ours enrolled Asian populations as well.

Compared with the above-mentioned studies, our meta-analysis has the advantages of a greater sample size and more comprehensive analyses by dividing the subjects into different subgroups on the basis of ethnicity and source of control. However, some aspects might affect the accuracy of our findings, such as the relatively small number of included studies, the lack of further subgroup analyses based on age, smoking status and other risk factors, as well as the absence of consideration of potential collective effects of our studied polymorphism with other relevant elements on the risk of BC. Therefore, the outcomes from the present study still need to be applied with prudence.

In conclusion, our findings do not support the assumption that *CCR5* Δ32 polymorphism has independent influences on individual susceptibility to BC. Nevertheless, these results are required to be further verified by studies with larger sample size in future.

## MATERIALS AND METHODS

### Literature searching

A systemic search was conducted in the databases of PubMed, EMBASE, Google Scholar Web and CNKI for all relevant papers published in English or Chinese language, using the combination of the following keywords: “chemokine C-C motif receptor 5” or “CCR5” or “CD195”, “breast cancer” or “breast carcinoma” or “mammary cancer”, and “polymorphism” or “mutation” or “variant”. To maximize the number of included studies, the references of relevant articles were all manually checked to supplement the yield of database searching.

### Inclusion and exclusion criteria

Every included report must conform to the following criteria: (1) with a case-control design; (2) assessing the relationship between *CCR5* Δ32 polymorphism and BC risk; (3) using validated genotyping method; (4) stating sufficient information on genotype and/or allele distribution both in case and control groups; and (5) focusing on human beings. Papers failing to meet any one of the above standards were eliminated from the present meta-analysis. Besides, excluded publications also contained letters, commentaries, case reports and review articles. If more than one article included the same group of participants, the one with the largest sample size or published most recently was selected.

### Data extraction

Two investigators independently extracted primary information from all eligible studies using the same data table. Information to be recorded contained the following aspects: first author’s name, publication year, original country, ethnic descent, number of cases and controls, control source, genotyping method, frequencies of genotypes and/or alleles in cases and controls, and *P* value for Hardy-Weinberg equilibrium (HWE). Cross-check of each item extracted from the eligible studies was completed by these two investigators to guarantee the accuracy of the data. If any discrepancies occurred, they would be settled through discussion between the two investigators until reaching a consensus.

### Statistical analysis

The intensity of the relationship of *CCR5* Δ32 polymorphism with BC susceptibility was appraised through calculating odds ratios (ORs) with the corresponding 95% confidence intervals (95% CIs). Between-study heterogeneity was examined with chi-square-based Q test. When *P* value less than 0.05 represented the existence of significant heterogeneity, the random-effects model was selected to calculate the overall ORs; otherwise, fixed-effects model has been selected. Sensitivity analysis was completed through removing each of included studies in turn and re-calculating pooled ORs to observe the impact on whole results. Additionally, Begg’s funnel plot and Egger’s regression test were both applied to inspect potential publication bias across selected studies. All data syntheses in the present meta-analysis were conducted with STATA 12.0 software (Stata Corporation, College Station, TX, USA).
